# Research Progress and Prospects of Saponins in the Treatment of NAFLD: A Narrative Review

**DOI:** 10.3390/molecules30214247

**Published:** 2025-10-31

**Authors:** Shuang Xue, Qiao Wang, Xuan Guo, Xingtong Chen, Yunyue Zhou, Jinbiao Yang, Yukun Zhang, Wenying Niu

**Affiliations:** School of Basic Medical Sciences, Heilongjiang University of Chinese Medicine, Harbin 150040, China

**Keywords:** saponins, non-alcoholic fatty liver disease, research progress, lipid metabolism, inflammation, oxidative stress

## Abstract

Non-alcoholic fatty liver disease (NAFLD) represents a prevalent chronic hepatic disorder worldwide, with its incidence continuing to rise in recent years. At the core of its pathological progression lie multiple interconnected mechanisms, including dysregulated lipid metabolism (e.g., abnormal accumulation of triglycerides in hepatocytes), impaired insulin sensitivity (which exacerbates hepatic lipid deposition), excessive production of reactive oxygen species (ROS) leading to oxidative stress, and sustained low-grade chronic inflammation that further amplifies liver tissue damage. Saponins have emerged as a crucial research direction for NAFLD intervention due to their advantage of multi-target regulation. This review synthesizes the mode of action of commonly studied saponins, including triterpenoid saponins and steroidal saponins: they regulate lipid metabolism by inhibiting fatty acid synthesis; modulate the gut microbiota; scavenge reactive oxygen species (ROS); alleviate endoplasmic reticulum stress; exert anti-inflammatory effects by inhibiting inflammasomes; and simultaneously regulate autophagy, restrain the activation of hepatic stellate cells, and modulate the gut microbiota, thereby achieving anti-apoptotic and anti-hepatic fibrosis effects. In conclusion, saponins can synergistically intervene in NAFLD through multiple mechanisms with good safety, while low bioavailability constitutes the main bottleneck for their clinical translation. In the future, it is necessary to further optimize formulation processes to improve absorption efficiency and conduct high-quality clinical studies to verify their long-term efficacy and drug–drug interactions, thus providing a new possible direction for NAFLD treatment.

## 1. Introduction

Non-alcoholic fatty liver disease (NAFLD) denotes a clinicopathological state characterized by excessive fat buildup in the liver (where hepatocellular steatosis impacts over 5% of hepatocytes) when there is no history of alcohol use and no other definite factors causing liver injury. It is capable of not only advancing to liver cirrhosis and hepatocellular carcinoma but is also connected with a variety of diseases like cardiovascular and cerebrovascular ailments, peripheral vascular disorders, diabetes mellitus, and cholelithiasis. At the same time, it raises the likelihood of developing diverse malignant tumors, including colorectal cancer, breast cancer, and pancreatic cancer [1,2]. In clinical practice, lipid-lowering drugs, hypoglycemic drugs, hepatoprotective agents, and insulin sensitizers are used for the treatment of NAFLD. However, these drugs primarily act by improving hepatic steatosis and inflammation, with limited effects on intervening in the disease progression. Moreover, their long-term safety, efficacy, and patient compliance remain difficult to evaluate accurately. Saponins are glycosides constructed by triterpenoids or spirostane compounds serving as aglycones, and they are predominantly distributed in terrestrial plants [3]. In many Chinese herbal medicines, saponins serve as the primary bioactive components; for instance, ginseng, licorice, and platycodon all contain saponin components. Research has verified that certain saponins exhibit potential therapeutic effects in improving non-alcoholic fatty liver disease (NAFLD). As a narrative review, this article aims to summarize the preclinical research progress on the mechanisms of saponins in intervening in non-alcoholic fatty liver disease (NAFLD) by collating and analyzing relevant research. Owing to the methodological characteristics of narrative reviews, the literature selection in this article is mainly based on the research accumulation of the author team in this field and the tracing of core studies, which may lead to a certain selection bias and failure to fully cover all relevant research findings. Future studies can adopt the standardized procedures of systematic reviews to further improve evidence synthesis. It should be noted that although there is a large accumulation of existing reviews on natural products for NAFLD intervention, systematic integration of the mechanisms of saponins has not yet been conducted, and the coverage of their non-lipid metabolic pathways (such as inflammation regulation, endoplasmic reticulum stress, autophagy, etc.) is relatively limited. Based on this, the core supplementary value of this review is reflected in three aspects: first, focusing on the subfield of saponins to construct a multi-dimensional “metabolism–inflammation–stress” mechanism interaction network; second, integrating recent preclinical studies to clarify the differences in action targets and regulatory intensities among different subtypes of saponins (e.g., ginsenosides, diosgenin, astragaloside IV, etc.); third, strengthening the translational application perspective in combination with the characteristic of low bioavailability of saponins, analyzing bottleneck issues in practical applications such as dose extrapolation and formulation optimization, and providing more targeted research and development suggestions.

Current studies remain constrained by limitations at the mechanistic level: for example, the specific mechanisms of action of saponins in targeting different subtypes of NAFLD (e.g., NAFLD with metabolic syndrome, isolated fatty liver) have not been clarified, and the investigation of novel regulatory molecules—including microRNAs (miRNAs) and long non-coding RNAs (lncRNAs)—remains relatively insufficient. Future research should focus on these areas and accelerate the advancement of clinical trials to clarify the potential value and application of saponins in the treatment of non-alcoholic fatty liver disease (NAFLD).

## 2. Saponins

**Figure 1:**
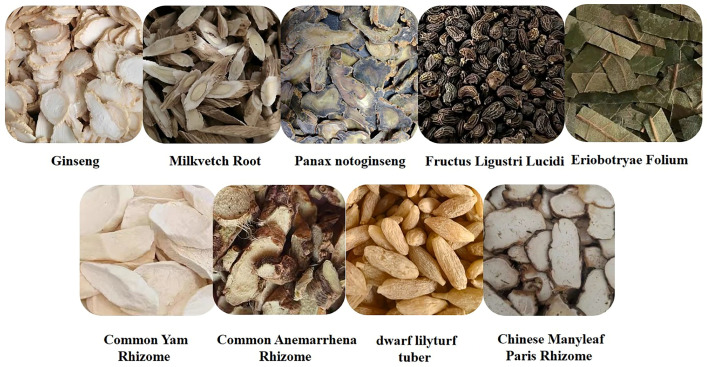
Saponins.

Saponins are secondary metabolites derived from plants, exhibiting notable diversity in both structure and function. Due to their varied functional characteristics and biological activities—encompassing both detrimental and beneficial effects—they have garnered growing interest in relevant research fields [4]. As illustrated in Figure 1, saponins represent a category of vital bioactive constituents in Chinese herbal medicines (CHMs), with a rich presence in plant sources like the rhizomes of ginseng, *Panax notoginseng* (san-shen), and polygonatum. Their biological activities cover immunomodulatory effects, anti-tumor actions, anti-inflammatory properties, protection against cardiovascular diseases, and blood glucose-lowering activity. Chemically, saponins are glycosidic compounds, where triterpenoids or spirostane compounds connect to sugar groups through glycosidic bonds. Both types are derived from oxidized polymers containing 30 carbon atoms; however, the difference lies in the fact that triterpenoid saponins retain 30 carbon atoms, while three methyl groups are removed from steroidal saponins. According to their aglycones, saponins can be categorized into triterpenoid saponins and steroidal saponins. Both types originate from the mevalonic acid pathway. The fundamental structure of triterpenoid saponins comprises 6 isoprene units and 30 carbon atoms, and they are mainly classified into tetracyclic or pentacyclic triterpenoids. Steroidal sapogenins, as spirostane derivatives, possess a six-membered ring structure with 27 carbon atoms; they are primarily divided into spirostanol-type steroidal saponins, isospirostanols, furostanols, and pseudospirostanols [5,6,7]. Most saponins exert effects in ameliorating non-alcoholic fatty liver disease (NAFLD), with the primary types involved being triterpenoid saponins and steroidal saponins. Below is a detailed introduction to representative saponins with NAFLD-modulating activity among their major characteristics.

### 2.1. Triterpenoid Saponins

#### 2.1.1. Tetracyclic Triterpenoid Saponins

Tetracyclic triterpenoid saponins include ginsenosides, notoginsenosides, astragaloside IV, gypenosides, hemsleyasaponins, mogrosides, and momordicosides, which have been the subject of extensive research. Among these saponins, ginsenosides and astragaloside IV are particularly prominent, as they possess the capacity to regulate lipid metabolism, modulate inflammation, and mitigate oxidative damage [8,9,10].

Ginseng (*Panax ginseng*), a perennial herb belonging to the Araliaceae family, is known as the “King of Herbs” [11]. Asian ginseng, Korean ginseng, and American ginseng have long been recognized as renowned medicinal plants with a history spanning thousands of years. To date, approximately 300 ginsenosides have been isolated and identified from different Ginseng species [12]. As the primary bioactive components of Panax species, ginsenosides can be classified into two categories—dammarane-type and oleanane-type—based on their chemical skeletons [13]. Among these, dammarane-type ginsenosides can be further divided into three subtypes, namely protopanaxadiol-type, protopanaxatriol-type, and ocotillol-type [14]. Over 90% of the total saponins that have been identified in ginseng roots originate from ginsenosides Rb1, Rb2, Rc, and Rd, protopanaxatriol-type Re, Rg1, and malonyl-ginsenoside derivatives [15]. Pharmacological studies have confirmed that ginsenosides exhibit diverse biological activities, covering multiple aspects such as anti-inflammatory effects [16,17], anti-cancer activity [18], cardiovascular protection [19], autophagy regulation [20], anti-apoptotic effects [21], and lipid metabolism modulation [22,23,24].

Astragalus (*Astragalus membranaceus*), a plant belonging to the genus Astragalus of the Fabaceae family, has thick and fleshy roots, which serve as its main medicinal part. As one of the most widely utilized Chinese herbal medicines, it was first documented in *Shennong Ben Cao Jing (Shennong’s Classic of Materia Medica)* [25]. Astragaloside IV serves as the primary bioactive metabolite of *Astragalus membranaceus* and also constitutes the key material basis underlying its pharmacological effects. It is a naturally occurring tetracyclic triterpenoid saponin made up of an aglycone segment and a sugar chain, featuring a definite molecular formula of C_41_H_68_O_14_ (which contains 41 carbon atoms, 68 hydrogen atoms, and 14 oxygen atoms). This distinctive structure confers upon it a broad spectrum of biological activities. Wild populations are largely distributed from Siberia to the Russian Far East, in addition to western and northern China. As a perennial species, it primarily thrives in temperate biomes [26,27]. The pharmacological actions of astragaloside IV encompass protection of the cardiovascular system [28], anti-fibrotic effects [29], anti-inflammatory effects [30], antioxidant activity, and regulation of glucose and lipid metabolism [31]. Owing to their extensive biological activities and medicinal potential, tetracyclic triterpenoid saponins (e.g., ginsenosides and astragaloside IV) are widely recognized as one of the most important types of saponins in natural product research and drug development.

Notoginseng (*Panax notoginseng*) is a perennial erect herbaceous plant and a well-recognized species of the Panax genus in the Araliaceae family, reaching a maximum height of 60 cm. Geographically, it is primarily distributed across Chinese provinces, including Yunnan, Guangxi, Jiangxi, and Sichuan [32,33]. Saponins are the main active components of notoginseng (*Panax notoginseng*). Approximately 200 chemical components have been isolated from notoginseng, including saponins, flavonoids, and cyclopeptides [34]. Dammarane-type triterpenoid saponins are the main bioactive components of notoginseng (*Panax notoginseng*). Notoginseng is a key herbal ingredient in many compound preparations, such as Compound Danshen Dripping Pills, Yunnan Baiyao, and Xuexuan Tongluo Capsules; it is also a common additive in health products, cosmeceuticals, and dietary supplements. Notoginseng saponins include ginsenosides (Rg1, Rb1, Rd), notoginsenosides (R1, A, B, C, D), and chikusetsusaponins (XVII, LXXV, IX). Among these, some are present in other Panax species, while others are unique to notoginseng. The types and quantities of these substances vary according to the plant’s age, growth conditions, and tissue types. Studies have indicated that variations exist in the types and contents of saponins between the aboveground parts (including flowers, stems, leaves, and fruits) and underground parts (comprising roots and rhizomes) of notoginseng [35], and notoginseng flowers (PNF) are rarely used [36]. Its extensive biological activities include antithrombotic effects [37], autophagy regulation [38], and anti-inflammatory effects [39].

#### 2.1.2. Pentacyclic Triterpenoid Saponins

Pentacyclic triterpenoid saponins represent a category of vital bioactive compounds found in plants, featuring diverse structures and a broad range of biological activities. They include glycyrrhizic acid, oleanolic acid, ursolic acid, betulinic acid glycosides, and friedelin derivatives. Among these pentacyclic triterpenoid saponins, oleanolic acid and ursolic acid have been the subject of extensive research regarding their effects in ameliorating NAFLD [40]. They are biosynthesized from 2,3-oxidosqualene (a precursor in primary sterol metabolism) via the mevalonate pathway. This precursor, 2,3-oxidosqualene, is transformed into dammarenyl cation by oxidosqualene cyclase. Subsequently, the dammarenyl cation undergoes ring expansion and cyclization to form the skeletons of α-amyrin and β-amyrin, which feature characteristic ring systems and methyl groups [41].

Oleanolic acid is a pentacyclic triterpenoid compound that is widely present in the plant kingdom. As a member of the oleanane family, this compound possesses eight chiral centers. It is not only commonly used as a medicinal material but also serves as an indispensable component of the human diet. Chemically, its structure is built around a pentacyclic framework, composed of six types of elements. Key structural features include a hydroxyl group at the C-3 carbon; two methyl groups at the C-4 and C-20 positions, respectively; one methyl group each at the C-8, C-10, and C-14 positions; a carboxyl group at the C-17 position; and a double bond between the C-12 and C-13 carbons. The stereochemistry of the -OH group at the C-3 position also plays a critical role in its physiological functions. Furthermore, the less common 3α-OH isomer demonstrates specific biological activities that are absent in the more widespread 3β-OH isomer [42]. It typically occurs in a nearly pure crystalline form within the leaves, grains, and fruits of olive trees and can prevent fungal attacks while acting as a defensive compound against herbivores or pathogens. As the conjugate acid of oleanolate, it is extensively distributed across a variety of plant species, existing either as triterpenoid saponin aglycones or free acids, and acts as a plant secondary metabolite [43]. Oleanolic acid has been used as a hepatoprotective drug, and there is clinical evidence supporting its efficacy in the treatment of hyperlipidemia. It exhibits a broad range of pharmacological and biological activities, and a number of clinical studies have illustrated the potential of oleanolic acid and its diverse derivatives in preventing or treating a range of diseases [44], including hepatoprotection [45], anti-cancer effects [46], anti-inflammatory effects [47], autophagy regulation [48], antioxidant activity [49], and modulation of lipid metabolism disorders [50].

Ursolic acid, also known as 3-β-hydroxyurs-12-en-28-oic acid, is a common secondary metabolite. It is naturally distributed across diverse plant species and falls under the category of ursane-type pentacyclic triterpenoids. Due to its safety and diverse biological activities, it has emerged as one of the most extensively researched ursane-type PTs (pentacyclic triterpenoids) [51]. As early as 1920, ursolic acid was first isolated and identified from the extract of apple epicuticular waxes. It contributes to the protection of apples against environmental stresses [52]. Its inherent chemical structure endows it with a series of unique biological activities [53]. In terms of its biological activities, it possesses a fundamental chemical structure that comprises five six-membered rings and exhibits effects such as hepatoprotection [54], anti-tumor activity [55], anti-fibrotic effects [56], anti-Parkinsonian effects [57], anti-apoptotic effects [58], and anti-anxiety effects [59]. It has been demonstrated to improve hepatic lipid metabolism through promoting fatty acid oxidation and suppressing adipogenesis [60]. Meanwhile, ursolic acid exhibits potent anti-inflammatory [61] and antioxidant properties [62].

### 2.2. Steroidal Saponins

Beyond triterpenoid saponins, steroidal saponins also constitute a category of important saponin compounds. They are a diverse group of compounds that are widely distributed in marine organisms such as starfish and sea cucumbers; in the plant kingdom, they are mainly distributed in families such as Asparagaceae, Dioscoreaceae, Liliaceae, Amaryllidaceae, Bromeliaceae, and Arecaceae. They also exist in large quantities in food crops. Common steroidal saponins include dioscin, timosaponin, ophiopogonin, paris saponin, yucca saponin, and avenacoside [63].

#### 2.2.1. Dioscin

Dioscin is a natural steroidal saponin, predominantly found in plants belonging to the Dioscoreaceae family. Its structural composition bears a high degree of similarity to that of endogenous steroids, such as cholesterol, progesterone, and estrogen. As the glycosidic form of diosgenin, dioscin is formed through the linkage of the trisaccharide α-L-Rha-(1→4)-[α-L-Rha-(1→2)]-β-D-Glc to diosgenin via a glycosidic bond—and it can be transformed into diosgenin through hydrolysis [64,65]. Diosgenin is the pharmacologically active form that exerts the main effects of dioscin, and it acts as a critical precursor for the synthesis of corticosteroids, including cortisone [66]. It exhibits a range of pharmacological activities, such as autophagy regulation [67], anti-inflammatory effects [68], cardiovascular protection [69], anti-Parkinsonian effects [70], anti-atherosclerotic effects [71], and anti-tumor activity [72].

#### 2.2.2. Timosaponins

In Traditional Chinese Medicine (TCM), *Anemarrhena asphodeloides* (known by its Chinese name “Zhimu”) boasts a long history of application in treating conditions such as arthralgia, hematochezia, tidal fever, and night sweats. Timosaponin AIII is a steroidal saponin and acts as one of the primary bioactive components of this plant (*Anemarrhena asphodeloides*) [73]. *Anemarrhena asphodeloides* is rich in saponins. Based on differences in aglycone structures, these saponins can be classified into spirostanol saponins (with a cyclic F-ring) and furostanol saponins (with an open-chain F-ring). Currently, timosaponin B-II (TB-II), timosaponin B-III (TB-III), and timosaponin A-III (TA-III) are the main research focuses [11]. These saponins display a range of pharmacological activities, with anti-tumor effects being one of their key properties [74], as well as anti-inflammatory effects [75], anti-obesity effects [76], antioxidant activity [77], and autophagy regulation [78]. To date, more than 40 types of timosaponins have been reported; as the most abundant saponin in *Anemarrhena asphodeloides*, it has become a key research focus in recent years [79].

#### 2.2.3. Ophiopogon Saponins

Ophiopogonin is a steroidal saponin derived from *Ophiopogon japonicus* (Maidong), a well-recognized Chinese herbal medicine that has long been valued for its health-promoting properties. Beyond its medicinal use, *Ophiopogon japonicus* is also a widely favored ornamental plant across East Asia. This plant is enriched with a diverse array of bioactive compounds, including dwarf lily tuber-13 (DT-13), ophiopogon-B (OP-B), ophiopogon-D (OP-D), and liriope-B (LP-B), among others. Specifically, OP-D is a rare C27 steroidal glycoside that is isolated from the tubers of *Ophiopogon japonicus* [80,81,82]. It displays a diverse range of pharmacological activities, with anti-inflammatory effects being one of its notable biological traits [83], as well as promotion of bone regeneration [84], anti-tumor activity [85], and improvement of pancreatic islet cells [86].

#### 2.2.4. Polyphyllin

Polyphyllins are the main active components of the medicinal plant Paris (*primarily Paris polyphylla var. chinensis)* and belong to the class of steroidal saponins—specifically, spirostanol-type steroidal saponins [87,88]. The dried rhizome of *Paris polyphylla* (a perennial herb belonging to the genus Paris in the Liliaceae family, first classified by Linnaeus) is a classic Traditional Chinese Medicinal (TCM) material. It boasts a long-standing medicinal history, particularly in Yunnan Province, and has been incorporated into every edition of the Chinese Pharmacopoeia to date [89]. It contains a variety of polyphyllin monomers, which are classified into four types: spirostanols, isospirostanols, furostanols, and pseudospirans. These monomers primarily consist of saponin-based bioactive components such as polyphyllin I, polyphyllin II, polyphyllin VI, formosanin C, polyphyllin VII, and yuanhuazosin, among others [90,91]. Their pharmacological effects include anti-tumor activity [92], treatment of ischemic stroke [93], promotion of ferroptosis [94], and alleviation of arterial hypertension [63].

As shown in Table 1, experimental results confirm that saponin compounds exhibit diverse biological activities, with their effective doses being clearly identified. These activities include autophagy regulation, anti-tumor effects, anti-inflammatory and antioxidant effects, lipid-lowering effects, anti-fibrotic effects, and hepatoprotective effects.

## 3. Mechanisms Underlying the Therapeutic Effects of Saponins on NAFLD

**Figure 2:**
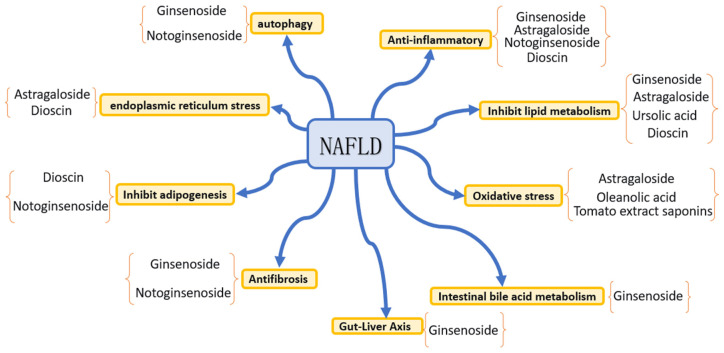
Mechanisms underlying the therapeutic effects of saponin compounds on NAFLD.

NAFLD is defined as a metabolic disorder that is triggered by the abnormal accumulation of triglycerides in hepatocytes. As depicted in Figure 2, its pathogenesis encompasses multisystem pathophysiological processes, such as dietary patterns, hepatocellular metabolic disorders, gut microbial communities, genetic susceptibility and epigenetic regulation, immune system dysregulation and inflammatory signaling cascades, as well as oxidative stress and endoplasmic reticulum stress [95]. As shown in Table 2, saponin compounds exert therapeutic effects on NAFLD through multiple pathways, mainly including anti-inflammatory effects, regulation of lipid metabolism, anti-oxidative stress activity, anti-fibrotic effects, suppression of lipogenesis, alleviation of endoplasmic reticulum stress, and regulation of autophagy, intestinal bile acid metabolism, and the gut–liver axis. The following text will summarize the main mechanisms of action of representative saponin compounds in intervening NAFLD that have been reported in recent years, with relevant con-clusions based on current preclinical research evidence.

### 3.1. Anti-Inflammatory Effects

Inflammation is a major pathological factor in the progression of non-alcoholic fatty liver disease (NAFLD), and hepatocyte death is one of the key causes triggering hepatic inflammation. As a defining characteristic of NAFLD patients, hepatic inflammation also serves as a critical driver for the progression of hepatic fibrosis. The liver, as an important organ for lipid metabolism, has its metabolic status closely associated with inflammatory responses; among these processes, macrophages play a vital role in the inflammatory response of NAFLD, which can participate in inflammatory processes by clearing pathogens and recruiting circulating inflammatory cells. Based on the close association between macrophage lipid metabolism and inflammation, dysregulated lipid metabolism in NAFLD may further promote inflammatory progression by affecting macrophage function. Therefore, regulating lipid metabolism may exert a potential therapeutic effect on the progression of NAFLD-related inflammation by reshaping the balance between M1 and M2 macrophages, and this direction holds significant therapeutic potential [96,97]. Macrophages polarize into separate phenotypes—specifically M1 and M2 macrophages—in response to stimulation by different damage-associated molecular patterns (DAMPs). In general, M1 macrophages exert pro-inflammatory effects, trigger liver damage, and can secrete significant quantities of pro-inflammatory mediators—including interleukin-1β (IL-1β), tumor necrosis factor-α (TNF-α), interleukin-6 (IL-6), nitric oxide (NO), and reactive oxygen species (ROS). In contrast, M2 macrophages predominantly secrete immunomodulatory factors, specifically interleukin-10 (IL-10) and interleukin-4 (IL-4). Their primary role is to establish an anti-inflammatory microenvironment and facilitate tissue repair in damaged organs, including the liver [10,98,99]. Lee, S.W. et al. [100], through in vivo and in vitro experiments, have confirmed that ginsenoside Rg3 exerts anti-inflammatory effects by inhibiting the adhesion between inflammatory cells and vascular endothelial cells and selectively improving the adhesion of inflammatory cells to liver sinusoidal endothelial cells (LSECs). Notably, Rg3-RGE treatment has no significant impact on the M1/M2 macrophage ratio or the expression of related genes. As the major non-parenchymal cells accounting for 15–20% of liver cells, LSECs play a crucial role in immune cell recruitment and hepatocyte adhesion by expressing chemokines and immune cell adhesion molecules. Studies have verified that Rg3-RGE treatment can inhibit lipopolysaccharide (LPS)-mediated activation of CiGEnC cells, reduce the adhesion between inflammatory cells and endothelial cells, and ultimately alleviate non-alcoholic fatty liver lesions through selective anti-inflammatory effects.

### 3.2. Inhibition of Lipid Metabolism

The liver occupies a pivotal position in lipid and lipoprotein metabolism, and metabolic disorders combined with perturbations in hepatic signaling pathways may drive the development of NAFLD [101,102]. Yang et al. [103] demonstrated that ginsenoside Rh4 exerts therapeutic effects on NAFLD by regulating bile acid metabolism (in both the liver and intestine), lipid metabolism, and phenotypes associated with hepatic inflammatory factors—all via the farnesoid X receptor (*FXR*) signaling pathway. The gut microbiota has been recognized as a key factor in the occurrence and development of metabolic diseases such as obesity and non-alcoholic fatty liver disease (NAFLD). Relevant studies have shown that NAFLD patients exhibit gut microbiota dysbiosis, specifically manifested by an abnormal ratio of Firmicutes to Bacteroidetes—an indicator that is often used to assess gut microbiota imbalance and obesity. Research has found that ginsenoside Rh4 can significantly reduce the abundance of intestinal bacteria, including Clostridium, Desulfovibrio, Campylobacter, Gammaproteobacteria, and Verrucomicrobia, while restoring the expression levels of gut immunity-related proteins (*GPR41*, *GPR43*, *GPR109A*). The above data suggest that ginsenoside Rh4 exerts a favorable regulatory effect on short-chain fatty acids (SCFAs), thereby improving hepatic lipid metabolism and intestinal immune function; in addition, it possesses the ability to regulate gut microbiota homeostasis and increase the levels of bile acids (BAs) and SCFAs. In summary, ginsenoside Rh4 can regulate bile acid metabolism-, lipid metabolism-, and liver inflammatory factor-related phenotypes in the liver and intestine through the farnesoid X receptor (*FXR*) signaling pathway, ultimately exerting a therapeutic effect on NAFLD. Toll-like receptor 4 (*TLR4*) is closely associated with hepatic steatosis and non-alcoholic fatty liver disease (NAFLD). Studies have demonstrated that the mRNA and protein levels of *TLR4* in the liver tissue of NAFLD patients are significantly higher than those in normal populations, while *TLR4* function-deficient mutant mice are resistant to diet-induced NAFLD. Liu et al. [104], through animal experiments, confirmed that astragaloside IV (AS-IV) can effectively improve dyslipidemia and alleviate hepatic steatosis in rats with non-alcoholic fatty liver disease (NAFLD) induced by a high-fat diet (HFD). Meanwhile, it downregulates the mRNA and protein expression levels of *TLR4*, *MyD88*, and *NF-κB p65* in the liver tissue of rats and reduces the serum levels of pro-inflammatory factors such as TNF-α, IL-6, and IL-8. These findings suggest that AS-IV exerts a mitigating effect on HFD-induced hepatic steatosis and NAFLD. However, this study has two limitations: first, it fails to clarify the specific molecular mechanism by which AS-IV inhibits the activation of the *TLR4*/NF-κB signaling pathway; second, the research is limited to animal models, and its conclusions still need to be further verified by in vitro experiments. Yao, H. et al. [105] demonstrated that dioscin exerts a significant effect on improving hepatic lipid metabolism and reduces triglyceride accumulation through the activation of the *Sirt1*/*AMPK* signaling pathway. *Sirt1* regulates energy metabolism by deacetylating *FOXO* and *PGC-1α*. Its role in obesity has been confirmed, and it is involved in metabolic regulation by modulating mitochondrial biogenesis, glucose, and lipid homeostasis. As an intracellular fuel sensor, *AMPK* can regulate energy balance and enhance *Sirt1* activity, with an interaction existing between the two. *AMPK* inhibits the cleavage and nuclear translocation of *SREBP-1c* to inactivate it, thereby alleviating hepatic steatosis and atherosclerosis while reducing lipid synthesis. In addition, *Sirt1* activates *AMPK* in an *SREBP-1c*-dependent manner and regulates downstream lipid metabolism-related proteins such as CPT and *FAS*. Therefore, *Sirt1*-mediated *AMPK* activation may serve as a novel mechanism or therapeutic target for combating NAFLD.

### 3.3. Oxidative Stress

As one of the most significant factors in NAFLD development, oxidative stress is fueled by factors such as decreased antioxidant enzyme activity and increased free radical concentrations in the organism. It occurs through the release of reactive oxygen species (ROS): when ROS interact with polyunsaturated fatty acids (PUFAs), they induce lipid peroxidation and generate aldehyde metabolites (e.g., malondialdehyde, MDA), ultimately leading to oxidative damage to cells. Liang et al. [106], through experimental studies, showed that astragaloside IV (AS-IV) can effectively inhibit hepatic lipid accumulation in non-alcoholic fatty liver disease (NAFLD)-model mice, while alleviating hepatic inflammatory responses and liver tissue damage. In palmitic acid (PA)-induced cell models, AS-IV treatment significantly reduces the intracellular levels of reactive oxygen species (ROS) and malondialdehyde (MDA) and markedly increases the level of glutathione peroxidase (GSH-Px). These results suggest that AS-IV can enhance cellular antioxidant capacity and mitigate cellular stress-induced damage, and its preventive effect on NAFLD may be achieved by inhibiting hepatic oxidative stress. Mhlindeli Gamede et al. [107] demonstrated that oleanolic acid administration, both with and without dietary intervention, exerts hepatoprotective effects in pre-diabetic conditions by reducing circulating triglycerides and preventing oxidative stress. This treatment was also shown to prevent structural changes in the liver, such as ballooning degeneration and inflammation. Zheng et al. [108] confirmed that ursolic acid (UA) can alleviate palmitic acid (PA)-induced lipid accumulation, mitochondrial dysfunction, and oxidative stress by regulating capric acid; it can also improve insulin resistance, a core pathogenesis of non-alcoholic steatohepatitis (NASH), through decorin (DCN)-mediated regulation of the insulin-like growth factor I receptor (*IGF-IR*) signaling pathway. Additionally, UA can relieve liver tissue hypoxia, which may be attributed to its inhibitory effect on the hypoxia-inducible factor 1 (*HIF-1*) signaling pathway. Furthermore, dioscin has been shown to reduce hepatic steatosis, inflammation, and oxidative damage, which in turn alleviates NAFLD. Yang et al. [109] demonstrated that tomatoside is extracted from cherry tomatoes, with salidroside A being identified as its primary saponin component. In mice, the NAFLD model established via administration of a high-fat diet (referred to as XT301) exhibited oxidative stress, lipid metabolism disorders, visceral fat deposition, and fatty liver—all of which could be mitigated by tomatoside (STE) treatment. The activation of the *Nrf2* signaling pathway is a crucial protective mechanism against oxidative stress. Under oxidative homeostasis, *Nrf2* is localized in the cytoplasm and regulated by *Keap1*, which inhibits its transmembrane transport. Under oxidative stress, *Keap1*’s ability to ubiquitinate *Nrf2* decreases; *Nrf2* accumulates stably, dissociates from *Keap1*, then translocates into the nucleus, binds to ARE, and initiates the transcription of antioxidant factors such as *SOD*, *HO-1*, and *NQO1*. STE can promote *Nrf2* nuclear translocation, a function that may only exist under oxidative stress conditions and is independent of *Keap1*. Meanwhile, STE can upregulate the expression of antioxidant factors, enhance the body’s antioxidant activity, and alleviate damage caused by a high-fat diet.

### 3.4. Anti-Fibrotic Effects

Hepatic fibrosis represents the most critical risk factor for hepatocellular carcinoma (HCC) and decompensated cirrhosis in patients with NAFLD. For individuals with NAFLD, age and comorbid conditions—such as hypertension, overweight, and diabetes mellitus—have been identified as confirmed risk factors for the progression of fibrosis [110]. Chen et al. [111] showed that treatment with ginsenoside CK or Rh1, either alone or in combination, can induce the apoptosis and inhibit the proliferation of hepatic stellate cells (HSCs). The activation of HSCs plays a crucial role in the fibrotic process. Ginsenoside CK and Rh1 inhibit HSC activation and reduce the expression of platelet-derived growth factor (*PDGF*) and transforming growth factor-β1 (*TGF-β1*), thereby suppressing the activation and proliferation of HSCs. Consequently, they exert potential hepatoprotective and anti-fibrotic effects in non-alcoholic fatty liver disease (NAFLD). Therefore, whether used as monotherapies or in combination therapies, ginsenosides and notoginsenosides may serve as promising candidates for alleviating liver injury or hepatic fibrosis. Li, N. et al. [112] demonstrated that ginsenoside Rg5 exhibits potent lipid-lowering activity in both in vivo and in vitro experiments. Specifically, ginsenoside Rg5 inhibits the activation of the *Notch1* signaling pathway by upregulating the expression of *Sirt1* protein, thereby suppressing hepatic lipid accumulation and fibrosis in non-alcoholic steatohepatitis (NASH). The anti-fibrotic effect of ginsenoside Rg5 stems from its inhibition of hepatic stellate cell activation, which in turn reduces collagen secretion. Experiments using a cellular fibrosis model have also demonstrated that it can decrease collagen fiber production by inhibiting the activation of LX2 cells.

### 3.5. Inhibition of Adipogenesis

A key histopathological hallmark of non-alcoholic fatty liver disease (NAFLD) is the accumulation of lipid droplets within hepatocytes. Therefore, it has long been hypothesized that lipids and lipid-derived compounds may act as disease drivers. Carrying a single nucleotide polymorphism (SNP) in the *PNPLA3* gene—specifically rs738409 (I148M)—raises genetic susceptibility to the development of NAFLD, and this protein is localized in the vicinity of lipid droplets within hepatocytes [113]. Xu et al. [114] demonstrated that notoginsenosides exert an influence on the key processes of fatty acid synthesis and oxidation, thereby regulating hepatic lipid flux. Specifically, *Panax notoginseng* saponin (PNS) can regulate the core processes of fatty acid synthesis and oxidation, thereby affecting hepatic lipid metabolic flux. It inhibits fatty acid production through synthetic genes such as acetyl-CoA carboxylase (*ACC*), fatty acid synthase (*FAS*), and sterol regulatory element-binding protein-1C (*SREBP-1c*) and promotes fatty acid decomposition via oxidative genes, including peroxisome proliferator-activated receptor α (*PPARα*), carnitine palmitoyltransferase 1 (*CPT-1*), and acyl-CoA oxidase 1 (*ACOX-1*). Under fatty acid-enriched conditions, PNS treatment enhances the oxygen consumption rate (OCR) of hepatocytes, which confirms its induction of high-level lipid oxidation. To clarify the mechanism by which *TLR4* inhibition reduces lipid deposition, the key energy sensor AMP-activated protein kinase α (*AMPKα*) was detected. It was found that PNS induces *AMPKα* activation to inhibit hepatocellular lipogenesis, and this effect can be blocked by *TLR4* activation. This suggests that PNS positively regulates *AMPKα* by inhibiting *TLR4*, thereby improving hepatic lipid metabolism.

### 3.6. Amelioration of Endoplasmic Reticulum Stress

Endoplasmic reticulum (ER) stress, which can be directly activated by glucolipotoxicity, also serves as a therapeutic target for NAFLD. Saturated fatty acids alter the phospholipid composition of the ER membrane and subsequently trigger ER stress via the sensors inositol-requiring enzyme 1α (*IRE1α*) and protein kinase R-like ER kinase (*PERK*)—even in the absence of unfolded proteins. These harmful stimuli, including ER stress, mitochondrial dysfunction, and oxidative stress in hepatocytes after lipid accumulation, can induce lipotoxicity, thereby leading to apoptosis, necroptosis, or pyroptosis [115]. Zhong et al. [116] showed that the expression levels of proteins related to the *PERK* and IRE1 pathways are significantly upregulated in the liver tissue of D-NAFLD rats, and administration of DIO can reduce their expression. Meanwhile, DIO can also downregulate endoplasmic reticulum stress-mediated apoptosis-related proteins such as *ATF4*, p-*CHOP*, and *caspase 12*. Furthermore, the inhibitory effect of DIO at a dose of 20 mg/kg on hepatic endoplasmic reticulum stress in rats is superior to that at 10 mg/kg. Zhou et al. [117] showed that hepatic endoplasmic reticulum (ER) stress can induce the activation of sterol regulatory element-binding protein-1c (*SREBP-1c*), thereby promoting lipogenesis and hepatic steatosis. Exposure of hepatocytes to free fatty acids (FFAs) significantly induces ER stress, which is manifested by increased expression of glucose-regulated protein 78 (*GRP78*), C/EBP homologous protein (*CHOP*), and phosphorylated protein kinase RNA-like endoplasmic reticulum kinase (p-*PERK*) and occurs synchronously with SREBP-1 activation and hepatic lipid accumulation. Astragaloside IV (AS-IV) can inhibit the activation of SREBP-1 in FFA-exposed hepatocytes and reduce the expression of the aforementioned ER stress markers to attenuate ER stress. In addition, AS-IV can alleviate ER stress in diabetic nephropathy both in vivo and in vitro. Its alleviation of FFA-induced ER stress in hepatocytes is achieved in an AMP-activated protein kinase (*AMPK*)-dependent manner, suggesting that *AMPK* negatively regulates lipid-induced ER stress in hepatocytes. In summary, astragaloside IV (AS-IV) can alleviate FFA-induced ER stress and lipid accumulation in hepatocytes by activating *AMPK*.

### 3.7. Regulation of Autophagy

The liver ranks among the most metabolically active organs in the human body, and autophagy fulfills a crucial role in both its hepatic physiology and pathology. At the cellular level, hepatic lysosomal/autophagic dysfunction is associated with pathogenic steatosis in the context of NAFLD. Both genetically and diet-induced obese mouse models are linked to impairments in hepatic autophagy and chaperone-mediated autophagy (CMA), which contribute to obesity-related hepatic steatosis and insulin resistance [118,119]. Lipotoxicity-induced endoplasmic reticulum (ER) stress exacerbates NAFLD and impairs normal autophagic processes. Autophagy also plays a role in regulating cell death and influencing cell survival; in the context of autophagy, mitophagy is specifically known to inhibit the production of reactive oxygen species (ROS)—substances that induce apoptosis. Numerous studies have demonstrated that in lipophilic cells, triglycerides in lipid droplets are degradable not just by cellular lipases, but also through a process termed lipophagy. During periods of enhanced lipophagy, lysosomal lipases mediate the breakdown of intracellular triglycerides and lipid droplets [120,121]. Choi, S.Y. et al. [122] demonstrated that Korean red ginseng extract (CRG) not only reduces lipotoxicity-induced cytotoxicity but also upregulates the expression of B-cell lymphoma 2 (*Bcl-2*). Lipotoxicity-induced endoplasmic reticulum (ER) stress exacerbates non-alcoholic fatty liver disease (NAFLD) and impairs normal autophagy. Autophagy is involved in regulating cell death and survival, among which mitophagy can reduce apoptosis by inhibiting the production of reactive oxygen species (ROS). *Coptis chinensis* rhizome extract (CRG) not only reduces lipotoxicity-mediated cytotoxicity but also upregulates the expression of B-cell lymphoma 2 (*Bcl-2*). This suggests that CRG can alleviate NAFLD by inducing *Bcl-2* expression to enhance mitophagy, and *Bcl-2* can inhibit cell death by counteracting lipotoxicity. In addition, CRG inhibits the mammalian target of rapamycin complex 1 (*mTORC1*) signaling pathway, exerting anti-steatotic effects by inducing mitophagy and anti-inflammatory activity through M2 polarization, respectively. Wang et al. [123] showed that *Panax notoginseng* saponin extract exerts anti-adipogenic, anti-fibrotic, and anti-inflammatory effects in a free fatty diet (FFD)-induced NAFLD mouse model. The study identified that the saponin extract is rich in ginsenosides Rh1 and Rg2—compounds that exert protective effects against NAFLD by inhibiting the NLR family pyrin domain-containing 3 (*NLRP3*) inflammasome, promoting mitophagy, and decreasing mitochondrial ROS (mtROS) production.

### 3.8. Other Mechanisms

The gut–liver axis describes the bidirectional crosstalk between the intestine and the liver. The liver shapes the composition and function of the gut microbiota and maintains the intestinal barrier by secreting bile acids (BAs) and inflammatory mediators into the bile ducts. In turn, the gut microbiota and their metabolites reach the liver via the portal vein, where they regulate bile acid synthesis as well as hepatic glucose and lipid metabolism. Dysregulation of the gut–liver axis is critical to the pathogenesis of non-alcoholic fatty liver (NAFL) and non-alcoholic steatohepatitis (NASH), manifesting as gut microbiota dysbiosis, intestinal barrier dysfunction, and heightened hepatic inflammatory responses [124]. As demonstrated by Yang et al. [103], Ginsenoside Rh4 may exert an interventional effect on non-alcoholic fatty liver disease (NAFLD) by regulating bile acid metabolism, lipid metabolism, and the phenotypes of hepatic inflammatory factors in the liver and intestine through the farnesoid X receptor (*FXR*) signaling pathway.

### 3.9. Synergistic Regulation and Pathway Crosstalk Among Mechanisms

The intervention of saponins in non-alcoholic fatty liver disease (NAFLD) does not act through a single pathway but forms a complex network effect via the cross-regulation of multiple mechanisms. Various pathways interact and cooperate synergistically in the pathophysiological process of NAFLD.

In the association between autophagy, inflammation, and fibrosis, saponins induce autophagic activation through the *AMPK*/*Sirt1* pathway. They eliminate damaged mitochondria via mitophagy to reduce the release of reactive oxygen species (ROS) and inhibit the activation of the *NLRP3* inflammasome and the secretion of pro-inflammatory factors such as IL-1β and TNF-α, thereby suppressing the activation and proliferation of hepatic stellate cells (HSCs) and delaying hepatic fibrosis [102,108,115]. Conversely, sustained inflammatory stress can inhibit autophagic flux, forming a vicious cycle of “inflammation–autophagy imbalance”. Preclinical evidence indicates that saponins can target this node to break the cycle and produce potential synergistic interventional effects.

In the crosstalk between lipid metabolism and oxidative stress, saponins inhibit de novo lipogenesis (DNL) through the *AMPK*-*ACC* pathway, while activating peroxisome proliferator-activated receptor α (*PPARα*)-mediated fatty acid oxidation (FAO) to reduce lipid droplet accumulation in hepatocytes. The alleviation of lipid overload can decrease endoplasmic reticulum (ER) stress and ROS production induced by lipotoxicity, thereby reversely improving mitochondrial function and restoring lipid metabolic homeostasis [103,106,111]. This may be the core synergistic mechanism underlying the improvement of hepatic steatosis observed in preclinical studies of saponins.

ER stress is closely associated with lipid metabolism and autophagy: when saponins activate the *AMPK* pathway to alleviate free fatty acid-induced ER stress, they simultaneously inhibit the activation of sterol regulatory element-binding protein-1c (*SREBP-1c*) to reduce fatty acid synthesis and improve the impaired autophagic flux, forming a positive regulatory loop of “ER stress alleviation–lipid metabolism improvement–autophagic function recovery”. Conversely, lipid overload exacerbates ER stress and disrupts autophagic balance, and the regulation of this pathway by saponins serves as an important supplementary mechanism for alleviating liver injury [104].

The linkage between anti-inflammation and lipid metabolism is also crucial: saponins inhibit the *NLRP3* inflammasome to reduce the secretion of pro-inflammatory factors, avoiding their abnormal regulation of key lipid metabolism enzymes such as acetyl-CoA carboxylase (*ACC*) and fatty acid synthase (*FAS*) [108]. Meanwhile, enhanced fatty acid oxidation can reduce lipid accumulation and the stimulation of immune cells by lipotoxicity, forming a synergistic “anti-inflammation–lipid metabolism optimization” effect to consolidate the interventional efficacy [103]. The aforementioned crosstalk among multiple pathways collectively constitutes the comprehensive pharmacological network of saponins in intervening in NAFLD and is also the core mechanism underlying their multi-dimensional improvement of the pathological process of NAFLD (Table 2).

**Table 2:** Pharmacological effects and mechanisms of saponins for NAFLD treatment.

Category	HCA	Mechanisms of Action	Classification of Mechanisms	Experimental Model	Dose Range	Ref.
Tetracyclic Triterpenoid Saponins	Ginsenosides	*VCAM-1*, *ICAM-1↓*	Inhibit inflammation	*C57BL/6 mice*	5 mg/kg	[100]
				*THP-1 cells*	25, 100 μg/mL, 24 h	
		*SREBP-1*, *ChREBP*, *LXR-β↓*, *Sirt1*, *LPL↑*	Inhibit hepatic lipid metabolism	*C57BL/6 mice*	60, 120 mg/kg	[112]
				*HepG2 cells*	0, 10, 20, 40, 50 μM, 24 h	
		*ZO-1*, *occludin*, *claudin-1↑*		*C57BL/6J mice*	60, 120, 180 mg/kg	[103]
		*mTORC1*, *Ccl2*, *Ccl5*, *Il-1β*, *Il-6*, *iNos*, *TNF-α↓*	Induce mitophagy	*C57BL/6N*	100, 300 mg/kg	[122]
				*Primary hepatocytes*	62.5 μg/mL, 24 h	
	Astragaloside	*GRP78*, *CHOP*, *p-PERK↓p-AMPK*, *p-ACC*, *p-SREBP-1c↑*	Inhibits lipid metabolism	*HepG2 cells*	50–200 μg/mL	[117]
		*TNF-α*, *IL-6*, *IL-8*, *TLR4 mRNA*, *MyD88 mRNA*, *NF-κB mRNA↓*	Inhibits inflammation	*SD rats*	20, 40, 80 mg kg^−1^ day^−1^	[104]
		*TNF-α*, *IL-6*, *5-LO*, *LTB4↓*	Anti-oxidative stress	*Kunming mice*	20, 40, 80 mg/kg	[106]
				*LO2*, *RAW264.7 cells*	20, 60, 100 μg/24 h	
	Notoginsenoside	*CD14↓* *TLR4↑*	Anti-adipogenesis	*C57BL/6J mice*	800 mg/kg/d	[114]
				*AML12 hepatocytes*	50 μg/mL,24 h	
		*IL-1β↓Tom40*, *Tim23*, *HSP60↓*	Inhibits inflammasome activation	*C57BL/6 mice*	50, 150 mg/kg	[123]
				*Primary hepatocytes isolated from C57BL/6 mice*	31.25, 62.5 μg/mL, 37 °c, 6 h	
		*PC-I*, *PC-III*, *TIMP-1↓*	Anti-fibrosis	*SD rats*	3 mg/kg/d	[111]
Pentacyclic Triterpenoid Saponins	Oleanolic Acid	*SREBP*, *TG*, *VLDL-C↓*	Anti-oxidative stress	*SD rats*	80 mg/kg	[107]
	Ursolic Acid	*IGF-IR*, *p-Akt↓p-Akt*, *HIF-1α↑*	Improves lipid metabolism	*C57BL/6J mice*	100 mg/kg/d	[108]
				*Primary hepatocytes isolated from C57BL/6J mice*	10 ng/mL	
Steroidal Saponins	Dioscin	*Sirt1*, *p-AMPK*, *p-SREBP-1c*, *CPT*, *FAS*, *SCD, FoxO1*, *ATGL↑*	Alleviates hepatic lipid accumulation	*C57BL/6J mice*	20, 40, 80 mg/kg	[105]
				*AML-12 hepatocytes*	600, 300, 150 ng/mL, 24 h	
		*p-mTOR*, *FASN*, *HIF-1α*, *RELA*, *VEGFA*, *p-mTOR↑IL-1β*, *TNF-α↓*	Inhibits lipid accumulation and inflammatory response	*SD rats*	50, 300 mg/kg/d	[125]
				*HepG2 cells*	5, 10, 20 μM, 24 h	
		*Bax*, *CytC*, *Apaf-1*, *caspase 3*, *caspase 9↑*	Alleviates endoplasmic reticulum stress	*SD rats*	10, 20 mg/kg	[116]
	Saponins from Tomato Extract	*AMPK*, *Nrf2↑* *FAS*, *SCD1↓*	Antioxidant	*C57BL/6 mice*	200 mg/kg/d	[109]

Note: ↑ Activation, ↓ Inhibition.

## 4. Bioavailability and Safety of Saponin Compounds

As shown in Table 3, the pharmacokinetics of ginsenosides, astragalosides, and notoginsenosides have been investigated in both animals and humans. These saponins are rapidly absorbed and readily distributed in tissues [126,127]. Astragalosides are primarily distributed in the liver, kidneys, lungs, spleen, and heart, with the highest concentrations being detected in the liver, kidneys, and lungs. Their distribution in the brain is extremely limited, a phenomenon that may be due to their difficulty in penetrating the blood–brain barrier [128]. Reports on the pharmacokinetics of oleanolic acid are scarce, yet it has garnered considerable interest. The effective delivery of oleanolic acid faces challenges, including poor water solubility, limited bioavailability, and the requirement for innovative drug delivery systems to boost its therapeutic efficacy. Notably, its oral bioavailability is extremely low—likely due to inadequate absorption and high metabolic clearance [129]—and its poor water solubility restricts both its bioavailability and therapeutic potential. Preclinical studies have demonstrated that oral ursolic acid is barely absorbed through the intestinal tract and is rapidly eliminated via hepatic metabolism following oral administration. Although intravenous injection of ursolic acid results in its systemic diffusion and non-specific distribution, the oral administration route is still regarded as more favorable than intravenous injection [130]. Dioscin is a poorly soluble drug, and oral administration is its main clinical route. However, pharmacokinetic studies have revealed that dioscin’s absolute oral bioavailability is merely 0.2%, accompanied by a long elimination half-life. Approximately 40% of dioscin can be degraded by gastrointestinal fluids, which may contribute to its low bioavailability. Both diosgenin and dioscin show low bioavailability—attributed to their poor absorption and slow metabolism—and this is likely to restrict their clinical application. To address this challenge, efforts should be dual-pronged: on the one hand, accelerate the development of new formulations of diosgenin and dioscin with high absorption and high bioavailability; on the other hand, synthesize other diosgenin derivatives with higher bioavailability by modifying the structures of diosgenin and dioscin [131]. Notably, saponins generally suffer from low bioavailability, which not only hinders the full exertion of their in vivo efficacy but also poses considerable challenges to the extrapolation of preclinically effective doses to humans. Based on the data presented in Table 1 and Table 2, the dose ranges of these compounds vary significantly across experimental models such as mice, rats, and different cell lines. The inherent variability of the models themselves has already increased the difficulty of interspecies dose conversion. Furthermore, low bioavailability leads to substantial differences in the absorption and distribution efficiency of drugs between humans and experimental models, which further enhances the uncertainty of dose extrapolation and makes it difficult to directly apply preclinically effective doses in clinical practice. Future studies should focus on overcoming this translational bottleneck by optimizing drug delivery systems (e.g., nanocarrier delivery technology) and conducting pharmacokinetic/pharmacodynamic (PK/PD) modeling, thereby improving the scientificity and reliability of their clinical translation.

## 5. Clinical Application Potential and Challenges of Saponins

As verified in previous animal and cell models, saponins have potential effects on non-alcoholic fatty liver disease (NAFLD). However, due to their potential toxicity, their clinical translation relies on human trial data to clarify the safe dose range. Given the scarcity of clinical research data on saponins for the treatment of NAFLD, this article summarizes the clinical data on the treatment of other diseases (see Table 4) to provide references for clinical medication [132,133,134,135,136,137,138]. Restricted by data availability, only results related to triterpenoid compounds were included. Among pentacyclic triterpenoid saponins (verified only as food), after optimizing the dosage form with edible vegetable oil, oral administration of 30 mg oleanolic acid (OA) showed no adverse reactions and improved bioavailability, solving the problems of solubility and biomembrane permeability of OA, which is a class IV highly hydrophobic compound in the Biopharmaceutics Classification System (BCS). Oral administration of 400 mg ursolic acid (UA) also caused no adverse reactions, laying a foundation for toxicity assessment in NAFLD-specific clinical trials [139,140]. In summary, existing studies have initially confirmed the clinical safety of some saponins, but there are still shortcomings in NAFLD-targeted toxicity research: a lack of long-term high-dose exposure data, unclear differences in toxicity from different plant sources, and unknown toxic reactions in NAFLD patients with special pathological conditions. Future studies should systematically explore their dose–toxicity relationships through NAFLD-targeted clinical trials to provide evidence for clinical translation. In addition, research on steroidal saponins such as diosgenin and anemarrhena saponin in the field of NAFLD is still in the preliminary stage, and their clinical translation potential needs further exploration.

## 6. Conclusions

In summary, saponin compounds exert a comprehensive therapeutic effect on non-alcoholic fatty liver disease (NAFLD) via a multi-target and multi-pathway approach. Their mechanisms of action mainly involve the following: regulating key enzymes related to hepatic lipid synthesis and breakdown, as well as the transcription factor sterol regulatory element-binding protein-1c (*SREBP-1c*), thus promoting fatty acid oxidation and inhibiting lipid accumulation; suppressing oxidative stress responses and inflammatory signaling pathways such as the nucleotide-binding oligomerization domain-like receptor pyrin domain-containing 3 (*NLRP3*) pathway to alleviate hepatocyte damage; regulating autophagic flux and the expression of apoptosis-related proteins to maintain hepatocyte homeostasis; and exerting anti-fibrotic effects. Furthermore, preclinical evidence indicates that these compounds can also indirectly improve intrahepatic metabolic disturbances in animal models by regulating the structure of intestinal microbiota and bile acid metabolic processes. These mechanisms are interrelated and synergistic, jointly forming a multi-dimensional pharmacological network of saponin compounds in the treatment of NAFLD. Among numerous saponins, ginsenosides and astragaloside IV have been studied more systematically. Their significant therapeutic effects have been confirmed in various NAFLD animal models, and their applications are relatively widespread. However, research on other saponins, such as some steroidal saponins (e.g., dioscin, timosaponin, etc.), in the field of NAFLD is still in the preliminary stage, and their specific targets and clinical transformation potential need further exploration. In the clinical practice of non-alcoholic fatty liver disease (NAFLD), patients often have comorbid metabolic diseases such as obesity, type 2 diabetes mellitus, and intestinal microbiota dysbiosis. These coexisting pathological conditions may affect the therapeutic response to saponins through multiple pathways. For example, obesity-related intestinal microbiota dysbiosis may interfere with the regulatory capacity of diosgenin on the endoplasmic reticulum (ER) stress pathway in the gut–liver axis, while insulin resistance induced by diabetes may weaken the effect of astragaloside IV on improving hepatic lipid metabolism via the AMP-activated protein kinase (*AMPK*) pathway. Since current mechanistic research on saponins in comorbid metabolic models is still limited, subsequent studies using NAFLD-complicated diabetes/obesity comorbid animal models and molecular interaction experiments are required to clarify the functional stability and comprehensive mechanisms of saponins in complex clinical scenarios. However, it is important to note that their clinical translation still faces key challenges: First, it is necessary to promote the standardization of research on saponin extracts, establish unified standards for the determination of active ingredient content and quality control, and ensure batch-to-batch consistency. Second, systematic preclinical pharmacokinetic studies should be strengthened. Combined with their low bioavailability characteristics, the administration routes and dosage form designs (such as nanocarrier delivery systems) of these compounds should be optimized to improve the effectiveness of in vivo exposure. Third, attention should be paid to potential herb–drug interactions, especially the risk of metabolic interference when combined with lipid-lowering drugs and hypoglycemic drugs, to provide a basis for rational clinical medication. Future studies should use these bottlenecks as breakthrough points and accelerate the advancement of high-quality clinical trials to truly realize the translational value of saponin compounds from basic research to clinical application. In conclusion, due to their pleiotropy and high safety, saponin compounds have become highly promising natural candidate drugs for the treatment of NAFLD.

## Figures and Tables

**Table 1:** Biological activities and pharmacological effects of saponin compounds.

Category	HCA	Structure	Source	Biological Activity/Application	Experimental Model	Dose Range	Ref.
Tetracyclic Triterpenoid Saponins	Ginsenosides	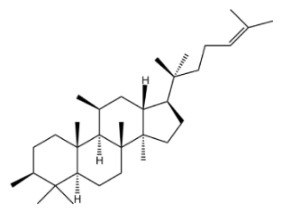	Asian Ginseng, American Ginseng, Sanqi Ginseng	Reduce hepatic steatosis	C57BL/6J mice	50/100 mg/kg/d	[16]
				Anti-inflammatory	HepG2, THP-1	2.5–10 μM, 48 h	[17]
				Autophagy	HepG2 cells	40 μmol/L	[20]
					C57BL/6 mice	130 mg/kg·d	
				Anti-cancer	C57BL/6J, BALB/C mice	60 mg/kg	[18]
					HCT116, CT26 cells	500 μM Rh4, 600 μM UDCA	
				Cardiovascular protection	C57/BL6 mice	25 mg·kg^−1^	[19]
				Anti-apoptosis	HHL-5 cells	0.2, 0.4 and 0.6 mM, 24 h	[21]
				Regulate lipid metabolism	SD rats	30, 60 mg/kg	[22]
	Astragaloside	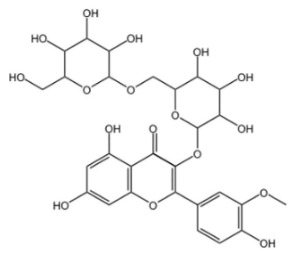	*Astragalus membranaceus*	Cardiovascular protection	C57BL/6J	40 mg/kg	[28]
					EMDM cells	100 μM, 4 h	
				Anti-fibrosis	C57BL/6J	0.2 mL/10 g	[29]
					BEAS-2B	10 μM AST, 12 h	
				Anti-inflammatory	SD	20, 40 mg/kg	[30]
					MPC5 cells	25, 50, 100 μM, 24 h	
				Antioxidant effect and regulation of glucose and lipid metabolism	Hepatocytes	100 μmol/L	[31]
	Notoginsenosides	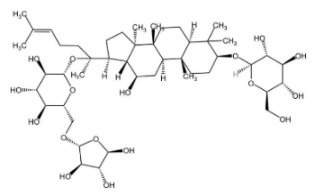	*Panax notoginseng*	Anti-thrombotic	C57BL/6 mice	75, 225 mg/kg^−1^	[37]
				Regulate autophagy	C57BL/6 mice	100 mg/kg	[38]
				Anti-inflammatory	C57BL/6J mice	20 mg/kg	[39]
Pentacyclic Triterpenoid Saponins	Oleanolic Acid	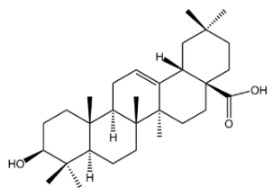	Apple, Glossy Privet Fruit	Hepatoprotection	C57BL/6 mice	300 mg/kg	[45]
					LO2 cells	10 mM,4 h	
				Anti-cancer	BALB/c nude mice	5, 10, 20 mg/kg	[46]
					HCT116, CT26, SW480 cells	80, 100, 120 μM	
				Anti-inflammatory	C57BL/6J mice	25, 50, 100 mg/kg	[47]
					HT22 cells	40 μM	
				Regulates lipid metabolism disorders	C57BL/6J mice	30, 60, 120 mg/kg/d	[50]
					HepG2 cells	100, 150 μM	
				Antioxidant	HaCaT cells	5–80 μM	[49]
				Regulates autophagy	C57BL/6J mice	6 mg/kg	[48]
				Hepatoprotection	C57BL/6 male mice	300 mg/kg	[45]
					L02 cells	2.5, 5, 10 μM	
	Ursolic Acid	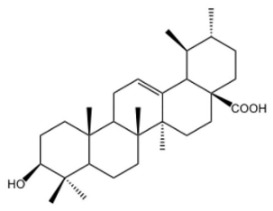	Marigold, Lavender	Hepatoprotection	C57BL/6J mice	25, 50, 100 mg/kg/d	[54]
					CD4+ T cells	0.2 μg/mL	
				Anti-tumor	BALB/c nude mice	200 mg/kg	[55]
					HFF-1, MRC5, Hep3B cells	10 mM	
				Anti-fibrosis	C57BL/6J mice	50 mg/kg/d	[56]
				Anti-Parkinson’s	C57BL/6 mice	10 mg·kg^−1^·d^−1^	[57]
					SH-SY5Y cells	2.5, 0.25 μM	
				Anti-apoptosis	C57BL/6J mice	50, 100 mg/kg	[58]
					EMSC	2.5, 5 μM	
				Anti-anxiety	Male Swiss mice	0.1, 1, 10 mg/kg	[59]
				Anti-inflammatory	WT, Nox2^−/−^, *NLRP3*^−/−^ mice	50 mg/kg	[61]
				Antioxidant	C57BL/6J mice	40 mg·kg^−1^	[62]
Steroidal Saponins	Dioscin	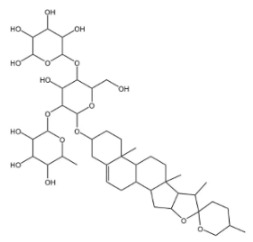	*Dioscorea opposita* Thunb, *Dioscorea bulbifera* L.	Regulates autophagy	Hailinge Brown Chicken	100, 500, 1000 mg/kg	[67]
					LMH chicken cells	0.3 mM	
				Anti-inflammatory	Wistar rats	60 mg/kg	[68]
				Cardiovascular protection	HL-1 cells	100, 200, 400 nM	[69]
					C57BL/6 mice	20, 40, 80 mg/kg	
				Anti-Parkinson’s	C57BL/6J mice	20, 40, 80 mg/kg	[70]
				Anti-atherosclerosis	ApoE^−/−^ mice	200 mg/kg	[71]
				Anti-tumor	NSCLC cell lines	5 μM, 72 h	[72]
	Anemarsaponins	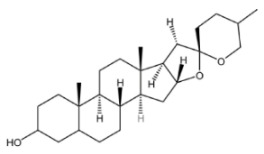	*Anemarrhena asphodeloides* Bunge	Anti-cancer	BALB/c nude mice	3, 6 mg/kg	[74]
					SW480, HCT116 cells	10 μM, 24 h	
				Anti-inflammatory	C57BL/6J mice	25, 50, 100 mg/kg	[75]
					BMDM cells	25, 50, 100 μM	
				Anti-obesity	C57BL/6J mice	10 mg/kg	[76]
					NCI-H716, 3T3-L1 cell lines	0–10 μM	
				Antioxidant	SD rats	0.1, 0.4 g/kg	[77]
				Regulate autophagy	GES-1, AGS, HGC27	0–5 μM, 24, 48 h	[78]
	Ophiopogonins	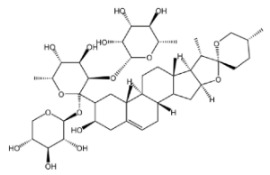	Hubei Ophiopogon Root	Anti-inflammatory	C57BL/6 mice	2.5, 5, 10 mg/kg	[83]
					A549 cells	0–20 μM	
				Promote bone regeneration	C57BL/6J mice	20 mg/kg	[84]
					HMEC cells	2.5, 5 μM	
				Anti-cancer	HHL-5, MHCC97-H cells	5, 10, 20, 40 μM, 24 h	[85]
				Improve islet cells	C57BL/6 mice	2.5, 5, 10 mg/kgd	[86]
					INS-1 cells	12.5, 25, 50, 60 μM	
	Paridis Saponins	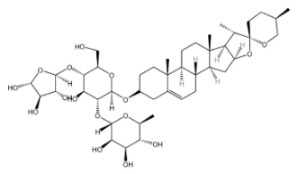	*Paris polyphylla* Smith	Anti-cancer	BALB/c nude mice	2.5, 5.0 mg/kg	[92]
					MKN-1, NUCC-3 GC cells	1.0, 2.0 μM, 48 h	
				Treat ischemic stroke	C57BL/6 mice	2, 5, 10 mg/kg	[93]
				Promote ferroptosis	C57BL/6J mice	10 mg/kg	[94]
					Cx3cl1, si-Hic1 cells	0, 0.25, 1.0, 2.0, 4.0 μM, 24 h	
				Alleviate pulmonary hypertension	SD rats	2 mg/kg	[63]
					FASMCs cells	2, 5, 10, 20, 30, 50 μM, 24 h	

**Table 3:** Bioavailability and safety of saponin compounds.

Category	HCA	Study Subjects	Bioavailability	Absorption Route	Peak Value	LD50 Value	Toxicology	Ref.
Tetracyclic Triterpenoid Saponins	Ginsenoside (C_48_H_82_O_18_)	Rats/Humans/ICR Mice	7.06%	Gastrointestinal Tract	Rats: T_max_ < 2 h Humans: T_max_ 1.19 ± 0.44 h Mice: T_max_ 0.4 ± 0.2 h	Rats: 750 mg/kg Mice: 200 mg/kg	Mice and rats: 5 g/kg, no toxicity after 2 years of oral administration; SD rats: 375 mg/kg/day, no toxicity after 6 weeks of oral administration; 5000 mg/kg, no toxicity after 2 years of oral administration.	[126]
	Astragaloside IV (C_28_H_32_O_17_)	Rats, Dogs	7.4%	Small Intestine	Rats: 3.78 mg/mL Dogs: 4.39 ± 2.59 mg/ml	800–1000 mg/kg	Rats: ≤200 mg/kg/day, no toxicity after oral administration.	[128]
Pentacyclic Triterpenoids	Oleanolic Acid (C_30_H_48_O_3_)	Rats	0.7%	Small Intestine	Rats: T_max_ 2–4 h Humans: T_max_ 2–6 h	>2000 mg/kg	Rats: ≤100 mg/kg/day, no toxicity after oral administration.	[129]
	Ursolic Acid (C_30_H_48_O_3_)	Rats	6%	Small Intestine	Plasma: T_max_ 2 h (C_max_ 184.73 ng/mL) Brain: T_max_ 2 h (C_max_ 325.2 ± 20.86 ng/g)	Oral: >2000 mg/kg Intraperitoneal injection: ~800 mg/kg	In animal experiments: <100 mg/kg/day, very safe, no obvious organ toxicity observed.	[130]
Steroidal Saponins	Dioscin (C_27_H_42_O_3_)	Rats/Humans	7%	Small Intestine	T_max_: 2–6 h	300–500 mg/kg	Rats were dosed at 300 mg/kg for 90 days; alanine aminotransferase increased, and liver function was impaired, showing a dose-dependent trend.	[131]

**Table 4:** Clinical research on saponin compounds.

Category	HCA	Drug	Subjects	Disease	Experimental Method	Route of Administration	Dosage	Incidence of Adverse Reactions (Treatment Group)	Ref.
Tetracyclic Triterpenoid Saponins	Ginsenoside	Zhenyuan Capsule	18–65 years old, 195 people	Diabetes Mellitus	Randomized Clinical Trial	Oral	100 mg/d 4 weeks	17.4%	[132]
		Red Ginseng	18 -60 years old, 149 people	Chronic Stress	Randomized Clinical Trial	Oral	200 mg/d 3 weeks	0%	[133]
		Korean Red Ginseng	Postmenopausal women aged 46–69, 73 people	Aging in Postmenopausal Women	Randomized Clinical Trial	Oral	500 mg/d	No data	[134]
		Xinyue Capsule	18–75 years old, 1068 people	Stable Coronary Artery Disease after Percutaneous Coronary Intervention	Randomized Clinical Trial	Oral	150 mg/d 24 weeks	2 cases of palpitations	[135]
	Astragaloside IV		12 healthy males	Muscle Injury	Randomized Clinical Trial	Oral	4 mg/d 1 weeks	No data	[136]
	Notoginsenosides	XueShuanTong	3072 adults	Ischemic Stroke	Randomized Clinical Trial	Oral	120 mg/d 3 months	1.0%	[137]
		XueShuanTong	21–33 years old, 12 people	Ischemic Stroke	Randomized Clinical Trial	Intramuscular Injection	150 mg	0%	[138]
						Intravenous Injection			
Pentacyclic Triterpenoids	Oleanolic Acid	Functional Olive Oil	18–30 years old, 22 people		Double-Blind, Randomized Controlled Trial	Oral	30 mg	No data	[139]
	Ursolic Acid		18–35 years old, 230 people		Randomized Clinical Trial	Oral	400 mg	0%	[140]

## Data Availability

Data are contained within the article.
